# Implementing deep learning models for the classification of *Echinococcus multilocularis* infection in human liver tissue

**DOI:** 10.1186/s13071-022-05640-w

**Published:** 2023-01-24

**Authors:** Mihaly Sulyok, Julia Luibrand, Jens Strohäker, Peter Karacsonyi, Leonie Frauenfeld, Ahmad Makky, Sven Mattern, Jing Zhao, Silvio Nadalin, Falko Fend, Christian M. Schürch

**Affiliations:** 1 Department of Pathology and Neuropathology, University Hospital and Comprehensive Cancer Center Tübingen, Tübingen, Germany; 2 Department of Surgery, University Hospital and Comprehensive Cancer Center Tübingen, Tübingen, Germany

**Keywords:** Echinococcus, Deep learning, Histology

## Abstract

**Background:**

The histological diagnosis of alveolar echinococcosis can be challenging. Decision support models based on deep learning (DL) are increasingly used to aid pathologists, but data on the histology of tissue-invasive parasitic infections are missing. The aim of this study was to implement DL methods to classify *Echinococcus multilocularis* liver lesions and normal liver tissue and assess which regions and structures play the most important role in classification decisions.

**Methods:**

We extracted 15,756 echinococcus tiles from 28 patients using 59 whole slide images (WSI); 11,602 tiles of normal liver parenchyma from 18 patients using 33 WSI served as a control group. Different pretrained model architectures were used with a 60–20–20% random splitting. We visualized the predictions using probability-thresholded heat maps of WSI. The area-under-the-curve (AUC) value and other performance metrics were calculated. The GradCAM method was used to calculate and visualize important spatial features.

**Results:**

The models achieved a high validation and test set accuracy. The calculated AUC values were 1.0 in all models. Pericystic fibrosis and necrotic areas, as well as germinative and laminated layers of the metacestodes played an important role in decision tasks according to the superimposed GradCAM heatmaps.

**Conclusion:**

Deep learning models achieved a high predictive performance in classifying *E. multilocularis* liver lesions. A possible next step could be to validate the model using other datasets and test it against other pathologic entities as well, such as, for example, *Echinococcus granulosus* infection.

**Graphical Abstract:**

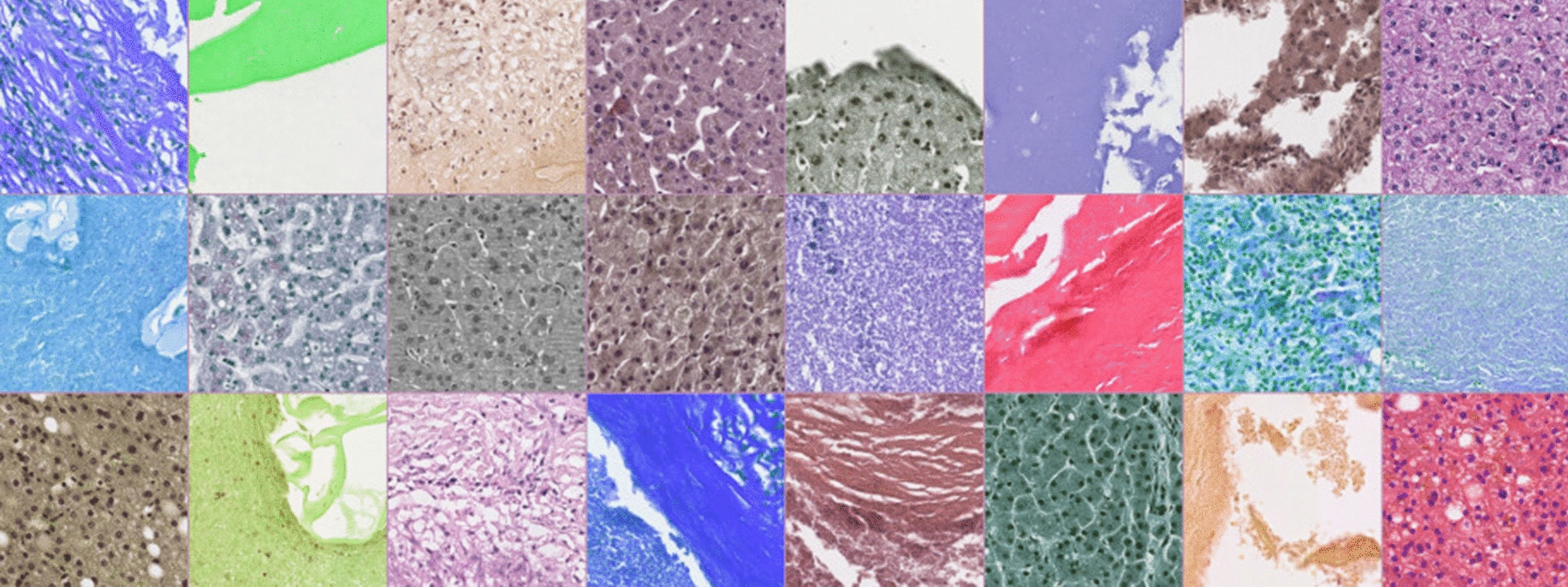

## Background

*Echinococcus multilocularis* is a cyclophyllid cestode causing life-threatening human infections. According to recent estimations, 18,235 (95% confidence interval: 11,900–28,200) new cases of alveolar echinococcosis (AE) occur annually, 91% of which occur in China, accounting for 666,434 disability-adjusted life years per annum [1]. Human infections may occur by accidental ingestion of eggs. The metacestode larvae reside usually in the liver, infiltrating the liver parenchyma. The pattern of tissue invasion with exogenous budding of the germinative layer and the alveolar appearance are very similar to a neoplastic process [2]. The infection may also spread to the lungs and brain [3]. Treatment is usually difficult, and the mainstay of therapy is surgery [4, 5]. Thus, histological examination plays an important role not only in the diagnosis, but also in defining surgical margins and the extent of lesions. However, the histological diagnosis, especially on the species level, can be very challenging. Since patients are often treated in specialized centers, histologic expertise can be problematic outside of these reference centers. Thus, a histologic decision-support system would be desirable.

Deep learning (DL) is composed of multiple processing layers to learn data representation [6]. These models have drastically improved speech recognition, visual object recognition and object detection among other fields [6]. The application of histologic DL methods are increasingly being studied in the field of oncology [7–10], but to our knowledge they are not yet applied to tissue-invasive parasitic diseases. The aim of this study was to test DL neural network models to classify *E. multilocularis* liver lesions from normal liver tissue. This represents a first step towards implementing existing DL histological frameworks used primarily in the field of oncology [11] to the histology of a parasitic disease and evaluate the model performances and address possible issues arising from the different morphology.

## Methods

### Patients and data

Histological tissue sections from a cohort of 28 patients with liver AE diagnosed between 2004 and 2021 were retrieved from the archive of the Institute of Pathology, University Hospital Tübingen. Normal liver tissue from 10 transplant donors from 2014 to 2021 were used as healthy controls. Non-infected adjacent liver tissues from eight of the echinococcosis patients were used as additional controls. In total, 59 echinococcus-infected and 33 normal/echinococcus-free liver tissue sections were used in this study. The study was approved by the local Ethics Committee of the University Hospital Tübingen (No. 017/2022B02).

### Analysis

#### Image processing, tiles, augmentation and normalization

Tissue sections were scanned in brightfield using a NanoZoomer 2.0HT_X scanner (Hamamatsu Photonics K.K., Shizuoka, Japan) at 20× magnification, and WSIs were stored as TIFF files. Areas containing the echinococcus laminar layer, germinative layer and fibrous capsule, as well as surrounding granulomatous inflammation were used for annotations. WSIs were manually annotated using QuPath [12]. A Conda environment was created as detailed by Berman et al. [11]. WSI images were randomized in a 60–20–20 ratio to training-validation-test sets and then broken down to 500-pixel tiles. The tissue detector function of PathML, a Python library for performing pre- and post-processing of WSIs, was run to detect tissue and separate it from the background and from various artifacts. The results were plotted and visually checked and compared to Otsu and triangle methods [11].

Suitable tiles were extracted as described by Berman et al. [11]. A strong data augmentation was performed with hue and saturation changes. Means and standard deviations were calculated (to normalize the tiles later). Cumulative tile numbers were displayed to decide whether the process was balanced or not. Suitable tiles were then normalized and re-sized accordingly.

#### Modeling

We applied three different model DL architectures, namely the VGG19 convolutional neural network (CNN) model with batch normalization, Squeezenet and ResNet18, all of which were pretrained on the ImageNet dataset [13, 14]. We set the output features to 2 according to our classes (echinococcus and normal liver). Our model was trained with a batch size of 48 with 15 epochs. We used cross entropy loss as a criterion and SGD (stochastic gradient descent) optimizer. Models were evaluated with entropy loss, accuracy, weighted accuracy, weighted precision, weighted recall and weighted f1 value. The best models (based on best epoch accuracy on the validation dataset) were saved and used for further analysis. Learning curves were plotted displaying entropy loss and weighted accuracy.

To identify the best tile-level probability cut-off (to decide whether a tile should be classified as echinococcus or normal when reaching the maximum tile-level accuracy), we inferred our trained model on the validation data set, and then applied the cut-off to make predictions on the test set.

Tile-level probabilities were visualized for the validation dataset to visually check the results of model training (to decide whether the model learned the important features or not).

Then, to generalize the tile-level predictions to whole slides (to simulate a real-life situation without annotations), we counted the tiles above our identified probability threshold to have a tile count above the boundary to calculate and plot area-under-the-curve (AUC) values.

Additionally, we used the gradient-weighted class activation mapping (GradCAM) method [15] to understand which regions/structures play an important role in the classification task. Superimposed attention-based heat maps were generated on the tile-level and inspected visually.

All computations were performed in Python 3.7.12 using the PathML framework [11] in Pytorch 1.4.0. All training was performed on a cloud server, using an Nvidia Tesla T4 GPU and an Intel® Xeon 14-core central processing unit (CPU) with 64 GB RAM (Intel Corporation, Santa Clara, CA, USA).

## Results

For the training dataset, 15,756 echinococcus tiles and 11,602 normal liver tissue tiles were extracted. The tissue detector successfully identified artifacts on the slides and liver tissue, but it tended to identify amorph fibrous material surrounding the laminated layer as an artefact, also reducing the number of echinococcus tiles. However, it was still the best-performing method when compared visually with the Otsu and triangle methods.

**Fig. 1:**
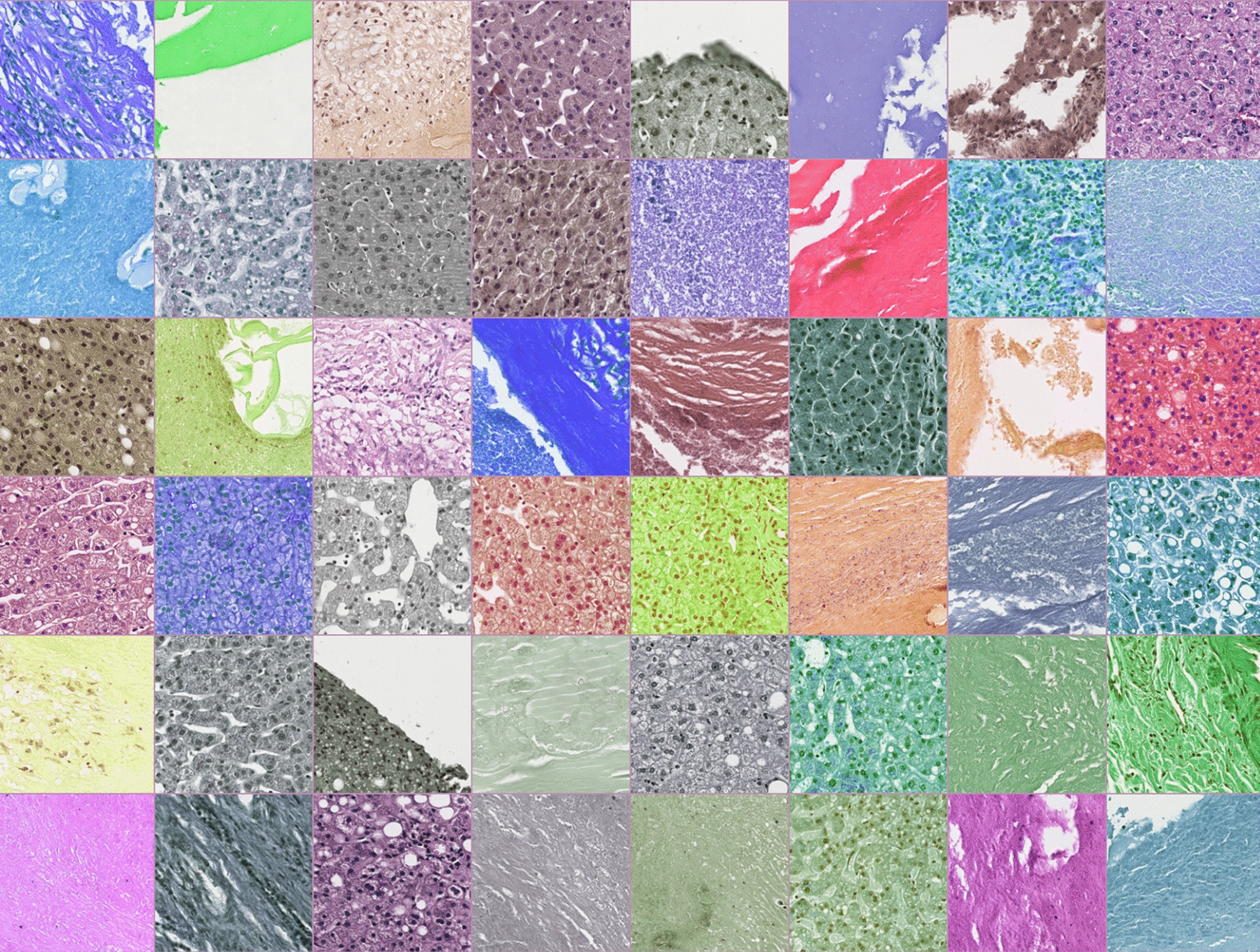
A sample training batch with 48 randomly selected augmented tiles from the training set, including hue and saturation changes

A sample batch is depicted in Fig. 1 showing the augmented extracted tiles of a 48-batch size used for training.

**Fig. 2:**
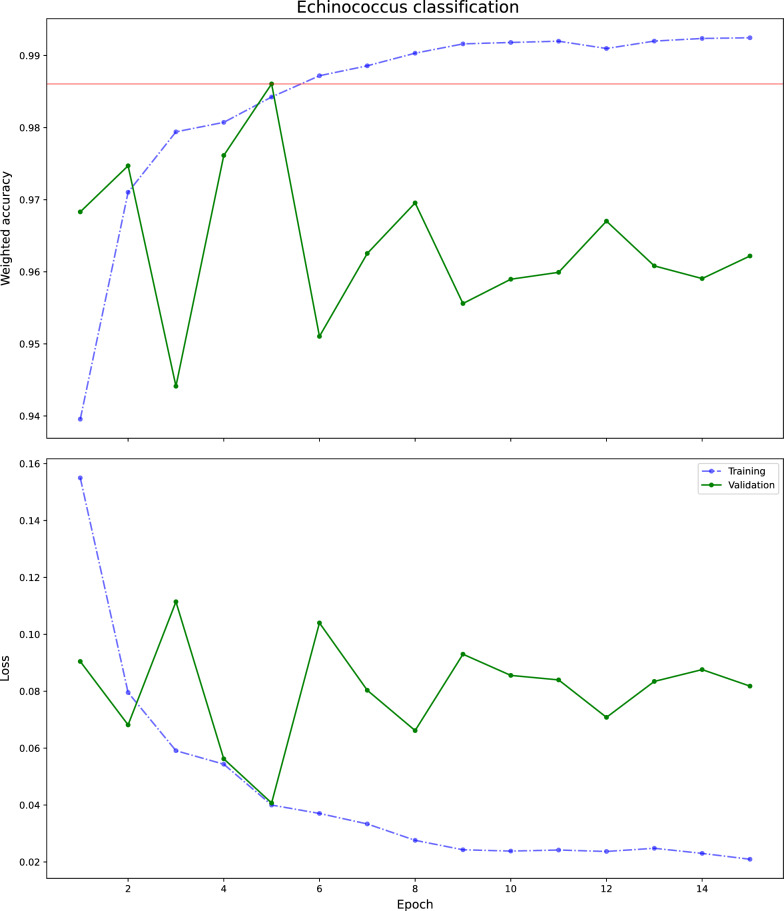
Learning curve of the ResNet-18 model

**Fig. 3:**
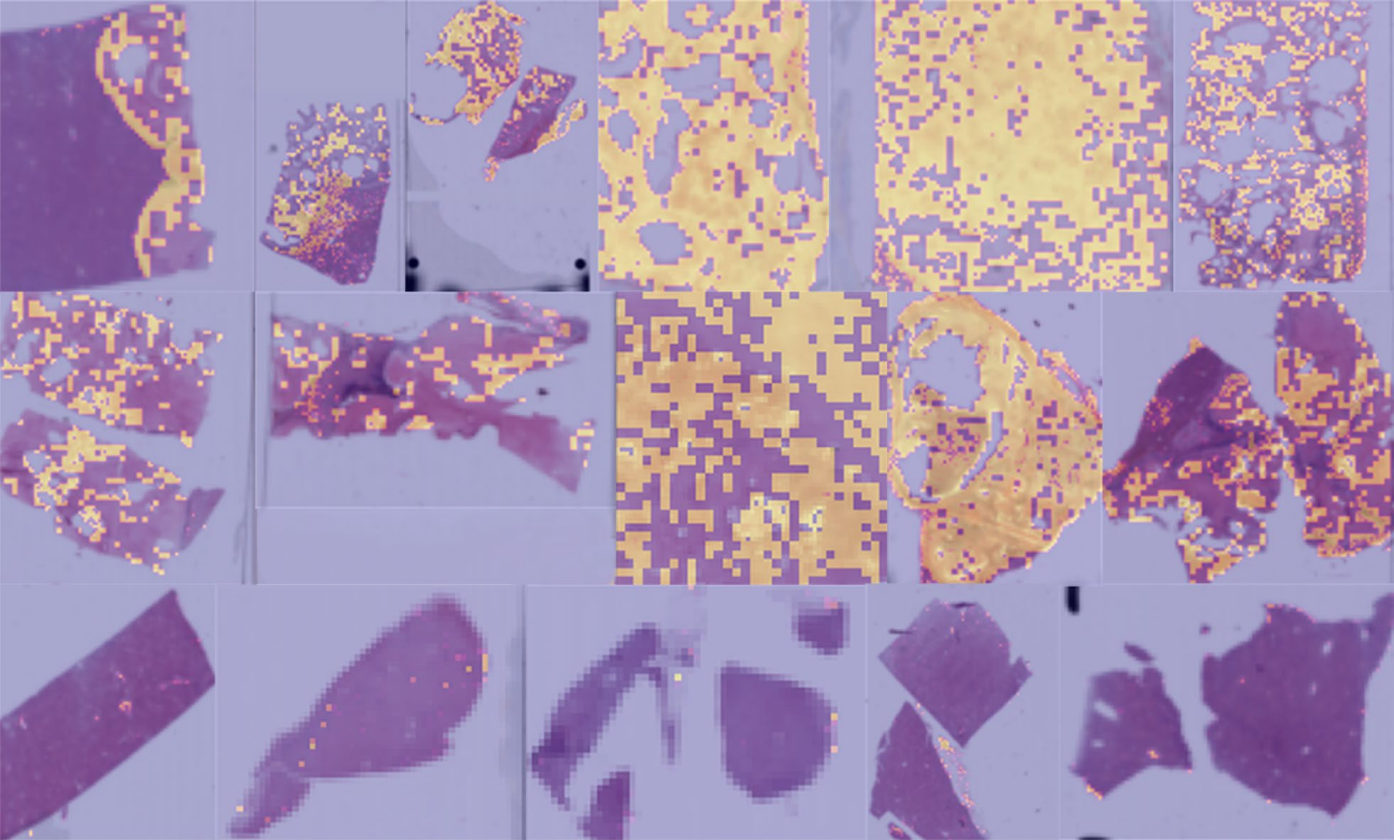
Tile-level prediction heat maps of the VGG19_bn model for the selected test set of echinococcus and normal whole-slide images. The yellow areas indicate a higher probability of echinococcus infection. Upper two rows of each image: echinococcus slides; the lower row of each image: control slides

Training the VGG19_bn model resulted in the best measures at the fifth epoch, namely an entropy loss of 0.054, accuracy of 0.98, weighted accuracy of 0.98, weighted precision of 0.98, weighted recall of 0.98 and weighted f1 of 0.98 on the validation dataset (model performances and other characteristics are shown in Table 1). The learning curve is depicted in Fig. 2. The best probability threshold for achieving the optimal tile-level accuracy was identified as 0.97. The best median test set accuracy at the applied echinococcus probability threshold was 0.94, (min–max: 0.3749–0.9998) (Table 2). On the slide level, the number of echinococcus tiles were tabularized (Table 3). Applying the cumulative echinococcus tile count per slide threshold (> 0.97 probability threshold), we obtained an AUC of 1.0 on the slide level. The Squeezenet [13]) and ResNet-18 models provided very similar performances (Table 1). Test set slide-level depiction of echinococcus probability is shown in Fig. 3.

**Table 1:** Comparison of model performance

Model performance parameters	DL neural models		
	VGG19_bn	Squeezenet	ResNet-18
Validation entropy loss	0.054	0.057	0.079
Validation accuracy	0.98	0.98	0.97
Validation weighted accuracy	0.98	0.98	0.97
Validation weighted precision	0.98	0.98	0.97
Validation weighted recall	0.98	0.98	0.97
Validation weighted f1	0.98	0.98	0.97
Median test set accuracy	0.94	0.92	0.9
Slide level test set AUC	1	1	1
Training time (in min) (15 epochs)	217	120	151

*AUC* Area under the curve,* bn* batch normalization, *DL* deep learning

**Table 2:** Slide-level accuracy of the test set of the different models

Slide_ID	Ground truth^a^	DL neural models		
		VGG19_bn	Squeezenet	Resnet18
0	0	0.974	0.991	1.000
1	0	1.000	1.000	1.000
2	1	0.818	0.819	0.839
3	1	0.956	0.932	0.946
4	1	0.937	0.919	0.901
5	1	0.798	0.683	0.792
6	1	0.806	0.799	0.823
7	0	0.998	0.997	0.997
8	1	0.487	0.505	0.475
9	1	0.817	0.808	0.850
10	1	0.822	0.818	0.833
11	0	0.997	0.999	1.000
12	0	0.999	0.999	1.000
13	1	0.948	0.934	0.943
14	1	0.593	0.581	0.578
15	0	0.995	0.995	0.998
16	1	0.375	0.392	0.371
17	1	0.717	0.718	0.703
18	0	1.000	0.999	1.000

^a^0: normal; 1: echinococcus

**Table 3:** Cumulative number of echinococcus tiles classified above the threshold per slide in the test data set with the different models

Slide_ID	Ground truth^a^	Tile count above threshold VGG19_bn	Tile count above threshold Squeezenet	Tile count above threshold Resnet18
0	0	3	1	0
1	0	2	0	0
2	1	1420	1410	1381
3	1	318	298	286
4	1	3230	3146	3052
5	1	488	686	494
6	1	1119	1158	1009
7	0	10	13	11
8	1	1997	1924	2027
9	1	2186	2100	2015
10	1	1161	1120	1135
11	0	6	2	0
12	0	3	2	0
13	1	259	288	256
14	1	1266	1326	1551
15	0	23	21	8
16	1	731	711	736
17	1	2560	2543	2533
18	0	1	3	0

^a^0: normal; 1 echinococcus

**Fig. 4:**
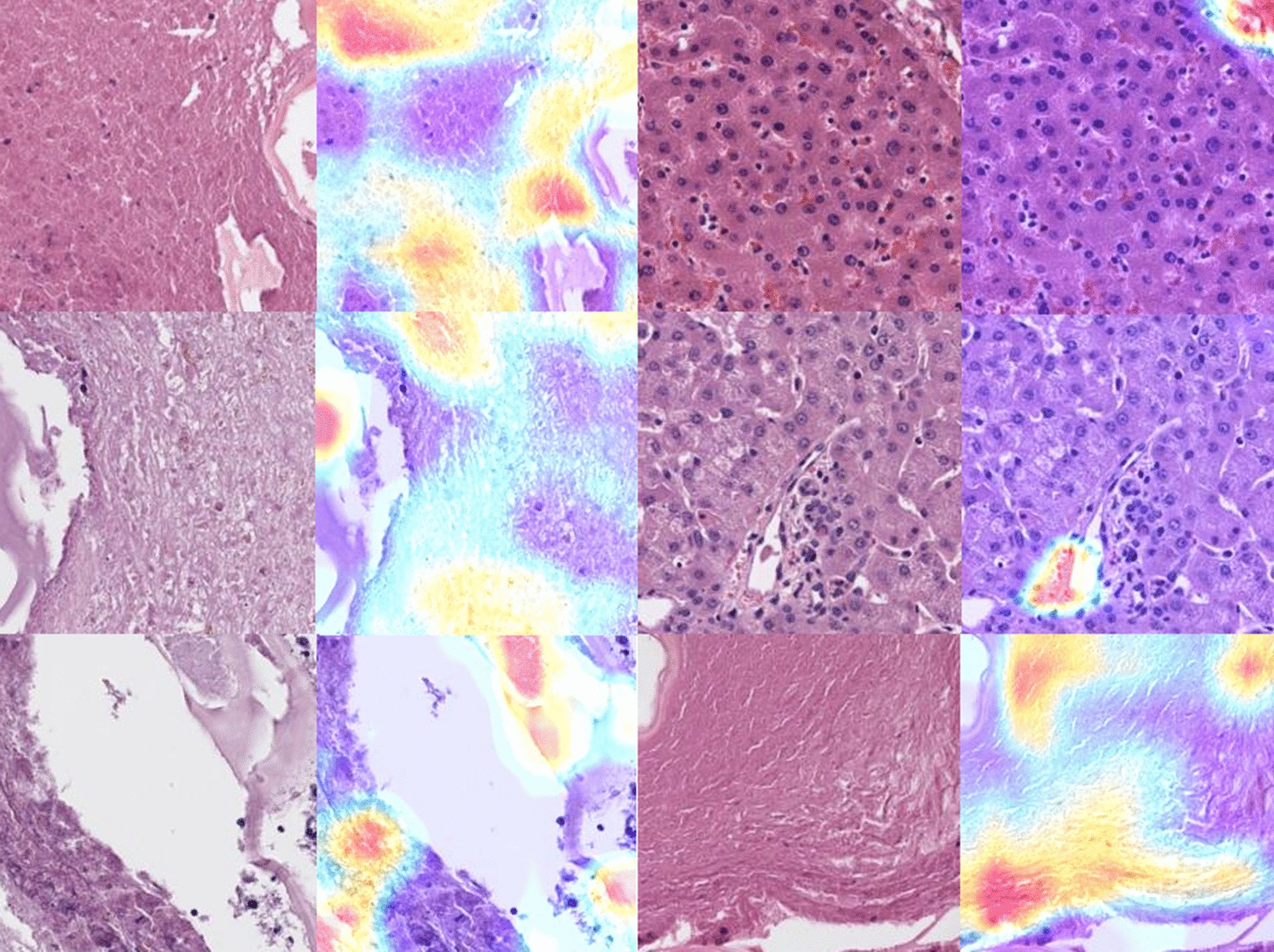
GradCAM heatmaps of some selected slides highlighting areas that play a more important role in the decision task than others. Red color indicates the areas with most importance. The upper 2 pictures in the right column show control liver tissue with portal tracts

As CNN classifiers are often criticized by the lack of human-interpretable transparency, several methods were developed to make the decision process more human-interpretable [16]. For example, the “attention-based” methods can identify regions that are relevant to the classification task. Thus, we applied one of them, the GradCAM method, to identify relevant regions/structures of interest [15]. The activation heatmaps in our study showed somewhat inconclusive results. In some tiles, the fibrous capsule played a more important role in the classification process than the parasitic structures, while in other tiles the opposite was true (Fig. 4).

## Discussion

The models we implemented showed a high accuracy in identifying *E. multilocularis* infection both at the tile level and on the slide level. The slide-level classification reached an AUC of 1.0 in the test dataset. The number of tiles classified as echinococcus positive varied greatly in the test set slides with some outliers with lower accuracy. Inspecting the predictions for the slides with lower accuracy did not reveal any reasons for the performance. The control slides with normal liver had a much lower number of echinococcus tiles if we applied the high tile-level probability threshold (mainly < 10 per WSI, in contrast to the several hundreds or thousands of echinococcus tiles of the echinococcus WSIs). Although we made no attempt to identify any cut-off value, the high level of difference between the echinococcus tiles is a promising finding.

The applied models were classifiers and not segmentation models. However, the generated slide-level heatmaps can also be applied to segment echinococcus lesions, as shown with the heatmaps of the validation and test datasets. Implementation to describe safety margins and resection status would be a promising avenue for future research. Nevertheless, the model learned to classify not just the laminated and germinative layers but also the surrounding fibrous capsule and the granulomatous tissue reaction. Thus, a low-level probability was also assigned sometimes to non-echinococcus structures, such as somewhat fibrotic portal fields and non-specific inflammatory alterations (Fig. 4).

Among the models, training the Squeezenet [13] model was the most time efficient (Table 1). Since no relevant differences were observed regarding model performance on the test set, we would favor the Squeezenet architecture over other models. This could be also efficiently trained using CPU-only machines that are probably more widely available than graphics processing units (GPUs). This could be an important point, since parasitic diseases are generally more frequent in developing countries where such resources are probably scarcer.

An important difference to neoplastic diseases is that echinococcus structures usually are much bigger than human cells, so using a lower resolution and bigger tile size is reasonable. This could decrease the amount of data to store and pre-process, which in turn reduces the training time. We believe that the configurations of different major parasitic structures and the extent of inflammatory response are probably more important than the chromatin morphology of the individual cells. This could be also confirmed to some degree by the GradCAM method [15].

To our knowledge, this is the first study to evaluate DL methods for the histological identification of a tissue-invasive parasitic disease. Applying DL methods to parasite detection and classification has an extensive literature, but mainly for apicomplexan organisms. Successful recognition of* Plasmodium* species in red blood cells (even smartphone-based) was recently reported [17–20]. A fuzzy cycle generative adversarial network was also successfully implemented to recognize *Toxoplasma gondii* parasites [17]. Regarding metazoa, DL image recognition is mainly applied to parasite-egg identification from stool samples [21, 22].

The only study we are aware of to apply DL for echinococcus recognition was conducted by Wu et al. using ultrasound images [23]. The authors used similar architectures (VGG19, ResNet18 and Inception-v3) as we did in the present study, and achieved a relatively high accuracy of 68.2–96% in classifying different types of ultrasound appearance of cystic echinococcosis. These values are quite similar to our results and offer a promising avenue for further investigation.

DL methods have a wide range of applications in histology, ranging from classification to object detection and segmentation. The leading field for such studies is oncology, most notably the frequent cancer types, including prostate [7], breast [8] and colon carcinoma. [9]. A popular direction of such studies is metastasis detection, such as the recognition of metastasis in (sentinel) lymph nodes [8, 10, 24], which is a time-consuming task in pathological routine diagnostics. While these tools are mostly used only as a decision support system to aid diagnosis, they can achieve a high accuracy comparable to that of a trained pathologist. It seems intuitive that similar methods could be exploited to other diseases as well, like AE which also exhibits an infiltrative growth pattern and metastatic capacity. As our results showed, DL methods can achieve a high predictive performance similar to that of models trained for oncologic tasks. Given the relative rarity of this disease, pathologists are not confronted with AE on a daily basis and the lack of experience may result in delays in diagnosis or other diagnostic errors. A well-trained model in case of high pre-test probability, such as radiological and clinical suspicion of AE, could probably aid the diagnosis in such a setting.

The main strength of our study was the successful implementation of a DL pipeline from Berman et al. [11], including data pre-processing to a tissue-invasive parasitic disease with the addition of GradCAM to identify decision relevant regions/structures. This also represents a limitation, since other processes with excessive fibrosis could cause false positivity, and did apparently also gain attention for the classification task. Furthermore, it could cause problems when defining resection margins since it does not necessarily indicate vital parasitic structures.

Given the relatively low number of patients, we used multiple slides per patient to provide a reasonably high data volume to train data-savvy architectures like VGG19. The slides from individual patients may exhibit a greater histological similarity to each other than to slides from other patients. This could lead to some bias towards an overestimation of model performance.

Our results should be further validated, if possible, with external data. A promising avenue for further research would be to involve *E. granulosus* cases as well and train the classification to separate it from cases of *E. multilocularis*. This can be a very difficult histological task sometimes, with no single reliable morphologic parameter in light microscopy slides. A previous multivariate analysis identified several factors, such as thickness and striation of the laminated layer and number and size of cysts [25]. Attention maps like GradCAM or other kinds of morphological marker identification can add a valuable input here. Immunohistochemistry can also separate the two species; however, these antibodies are only available in highly specialized laboratories. Thus, a simple classification for hematoxylin/eosin-stained slides would be desirable. Furthermore, training the applied models to classify other tissue-invasive parasitic diseases can be also listed as a future direction.

## Conclusions

Deep learning models achieved a high predictive performance in classifying *E. multilocularis* liver lesions. A possible next step could be to validate the model using other datasets and test it against other pathologic entities as well, such as *E. granulosus* infection.

## Data Availability

The script and the detailed results are available under the link: https://github.com/SchurchLab/Echinococcus. The WSI dataset is available from the corresponding author upon reasonable request.

## Abbreviations

AE: Alveolar echinococcosis
AUC: Area under the curve
DE: Deep learning
GradCAM: Gradient-weighted class activation mapping
WSI: Whole slide images
